# ^240^Pu/^239^Pu and ^242^Pu/^239^Pu atom ratios of Japanese monthly atmospheric deposition samples during 1963–1966

**DOI:** 10.1038/s41598-019-44352-7

**Published:** 2019-05-30

**Authors:** Yoshihito Ohtsuka, Michio Aoyama, Yuichi Takaku, Yasuhito Igarashi, Michinari Hattori, Katsumi Hirose, Shun’ichi Hisamatsu

**Affiliations:** 1 Department of Radioecology, Institute for Environmental Sciences, Rokkasho, Aomori, 039-3212 Japan; 2 Center for Research in Isotopes and Environmental Dynamics, Faculty of Life and Environmental Sciences, University of Tsukuba, Tennoudai 1-1-1, Tsukuba, 305-8572 Japan; 3 Institute for Integrated Radiation and Nuclear Science, Kyoto University, 2, Asashiro-Nishi, Kumatori-cho, Sennan-gun, Osaka, 590-0494 Japan; 4 Faculty of Science and Technology, Sophia University, Chiyoda-ku, Tokyo, 102-8554 Japan; 5 Institute for Environmental Sciences, Rokkasho, Aomori, 039-3212 Japan; 6 Present Address: Department of Psychiatry, Kochi Health Sciences Center, Ike 2125-1, Kochi, Kochi, 781-8555 Japan

**Keywords:** Environmental monitoring, Atmospheric chemistry

## Abstract

Global fallout plutonium isotopic ratios from the 1960s are important for the use of Pu as environmental tracers. We measured the ^240^Pu/^239^Pu and ^242^Pu/^239^Pu atomic ratios of monthly atmospheric deposition samples collected in Tokyo and Akita, Japan during March 1963 to May 1966. To our knowledge, our results represent the first data measured for actual atmospheric deposition samples collected continuously during the 1960s. Both atomic ratios increased rapidly from March 1963 to June 1963, followed by a gradual increase until September 1963. Then, both ratios declined with a half-life of approximately 5.6 months. The observed temporal changes of the ratios were likely caused by the upper-stratospheric input of nuclear debris from high-yield atmospheric nuclear weapon testing during 1961–62, followed by its downward transport to the troposphere.

## Introduction

**Figure 1:**
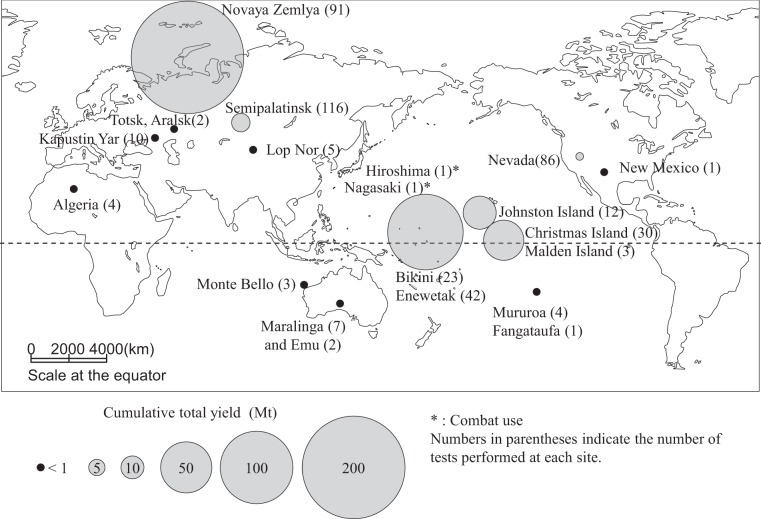
Atmospheric nuclear weapon test sites and cumulative total yields at each site (indicated by symbol size) during 1945–1966^27^. Four tests in the Pacific Ocean and three in the Atlantic Ocean are not included because of no description of accurate detonation sites^27^. Their cumulative yields are approximately 0.11 Mt. Background map is created using Adobe Illustrator CS6 (https://www.adobe.com/).

Most plutonium isotopes in the environment today are derived from artificial sources, primarily atmospheric nuclear weapon tests during 1945–1980, though an extremely small amount of ^239^Pu occurs naturally^1^. Atmospheric detonations of 502 nuclear devices with a total yield of 440 Mt (TNT equivalent) have occurred at the proving sites shown in Fig. 1, including the two combat uses in Hiroshima and Nagasaki. The former USSR performed nuclear testing mainly at high latitudes in the Northern Hemisphere, whereas the USA performed testing at low latitudes. Additional detonation tests were carried out by People’s Republic of China at middle latitude of Northern hemisphere and by France and the UK in the Southern Hemisphere. Plutonium introduced into the troposphere and stratosphere by these detonations was advected and diffused in the atmosphere before final deposition throughout the world^2^.

Various industrial and military accidents have dispersed a much smaller amount of Pu compared to nuclear weapon testing. Nuclear accidents at the Chernobyl nuclear power plant (NPP) in the former USSR in 1986^3^ and the Fukushima Dai-ichi NPP in Japan in 2011 dispersed artificial radionuclides including Pu^4^. A waste storage tank at the Mayak nuclear material production complex in the Chelyabinsk region in the former USSR had major leaks in 1957, and the leakage, containing Pu, flowed into the Techa River^5^. Furthermore, bomber aircrafts carrying atomic bombs crashed in Palomares, Spain in 1966^6^ and Thule, Greenland in 1968^7^, resulting in local Pu contamination.

Plutonium isotopic compositions differ depending on their origin. For example, the mean ^240^Pu/^239^Pu atomic ratio (±a standard deviation) of cumulative global fallout Pu is 0.18 ± 0.01 in surface soils collected from 24 sites in 31–70°N region^8^. ^240^Pu/^239^Pu atomic ratios above 0.18 were observed in soils around the Chernobyl^3,9^ and Fukushima Dai-ichi NPPs^4^, whereas the ^240^Pu/^239^Pu atomic ratios of spent nuclear fuels from civilian nuclear reactors vary based on the type of reactor and burn-up^10,11^. Ratios below 0.18 have been measured in soils around aircraft crash-sites^12,13^. Plutonium isotopic signatures can be used to identify the origin of Pu^14–16^ and as a tracer for studying the behaviour of Pu in the environment^17^.

Although the ^240^Pu/^239^Pu atomic ratio of cumulative global fallout Pu has been widely used to evaluate the origin of Pu, some researchers have reported temporal variations of the ratios in crop archives^11^, lake sediment cores^16,17^, and glacial ice core samples^11,18,19^. The ^240^Pu/^239^Pu atomic ratios of aerosol samples collected in the 1960s^20^ were not in agreement, likely due to differences in sampling locations, the representativeness of the samples, and analytical uncertainties. Despite this discrepancy, peaks in mid-latitude atmospheric concentrations and Pu deposition were observed in 1963, just after the peak of high yield atmospheric testing in 1962. Therefore, ^240^Pu/^239^Pu atomic ratios dating to the 1960s are important for the use of Pu atomic ratios as tracers. Although ^242^Pu/^239^Pu atomic ratios in atmospheric deposition has been scarcely reported, the ratio is useful for studying on the behaviour of Pu in the environment.

Here we report precise ^240^Pu/^239^Pu and ^242^Pu/^239^Pu atomic ratios in mid-latitude atmospheric fallout (wet + dry) samples collected in Japan during 1963–1966, as measured by multi-collector inductively coupled plasma mass spectrometry (MC-ICP-MS). Our results are discussed in the context of the atmospheric half-life of Pu transported in the stratosphere.

## Results

### Atmospheric deposition sample archives

The Meteorological Research Institute (MRI, Japan) has collected monthly atmospheric deposition samples with open surface samplers since April 1957 in Tokyo (35° 42′N, 139° 36′E) and since June 1959 in Akita (39° 43′N, 140° 06′E). Distilled water was put into samplers with effective surface areas of 0.5 or 1 m^2^. After each monthly collection (wet and dry deposition), the samples were evaporated to dryness, and the residues were dried at 110 °C. An aliquot of each residue was analysed for ^239 + 240^Pu by α-ray spectrometry, and the results were published as monthly ^239 + 240^Pu deposition rates^21–25^. Here, we analysed the ^240^Pu/^239^Pu and ^242^Pu/^239^Pu atomic ratios of aliquots of the remaining samples collected during March 1963 to May 1966, excluding April 1963 and March 1966 because these samples did not remained. The cumulative ^239 + 240^Pu deposition in Tokyo during March 1963 to May 1966 was reported to be 19.7 Bq m^−2^, which is about 40% of that during 1945–2000 (47.7 Bq m^−2^)^24,25^. Atmospheric deposition samples from both Tokyo and Akita from seven months (July, August, September, October, and November 1963 and November and December 1964) were analysed to compare between the two locations 450 km apart. For the other months, the analysed samples were collected in either Tokyo or Akita (Table 1).

**Table 1:** ^240^Pu/^239^Pu and ^242^Pu/^239^Pu atomic ratios of atmospheric deposition samples collected during March 1963 to May 1966 in Tokyo and Akita, Japan.

Collecting month	Atomic ratio^a^			
	^240^Pu/^239^Pu		^242^Pu/^239^Pu	
	Tokyo	Akita	Tokyo	Akita
Mar-1963	0.186 ± 0.001		0.0039 ± 0.0001	
Apr-1963				
May-1963	0.210 ± 0.001		0.0050 ± 0.0001	
Jun-1963	0.235 ± 0.001		0.0060 ± 0.0001	
Jul-1963	0.239 ± 0.001	0.235 ± 0.002	0.0062 ± 0.0001	0.0061 ± 0.0001
Aug-1963	0.239 ± 0.002	0.242 ± 0.002	0.0062 ± 0.0002	0.0062 ± 0.0002
Sep-1963	0.249 ± 0.002	0.245 ± 0.002	0.0064 ± 0.0001	0.0062 ± 0.0001
Oct-1963	0.241 ± 0.002	0.240 ± 0.002	0.0061 ± 0.0001	0.0059 ± 0.0002
Nov-1963		0.244 ± 0.002		0.0065 ± 0.0002
Dec-1963	0.238 ± 0.004	0.235 ± 0.002	0.0059 ± 0.0004	0.0057 ± 0.0001
Jan-1964		0.233 ± 0.002		0.0057 ± 0.0001
Feb-1964		0.230 ± 0.002		0.0054 ± 0.0001
Mar-1964		0.222 ± 0.002		0.0053 ± 0.0001
Apr-1964		0.215 ± 0.001		0.0050 ± 0.0001
May-1964		0.212 ± 0.001		0.0052 ± 0.0001
Jun-1964		0.214 ± 0.001		0.0048 ± 0.0001
Jul-1964	0.195 ± 0.002		0.0041 ± 0.0001	
Aug-1964	0.180 ± 0.001		0.0037 ± 0.0003	
Sep-1964	0.182 ± 0.001		0.0037 ± 0.0001	
Oct-1964	0.188 ± 0.001		0.0043 ± 0.0002	
Nov-1964	0.187 ± 0.001	0.193 ± 0.001	0.0039 ± 0.0003	0.0042 ± 0.0002
Dec-1964	0.192 ± 0.002	0.193 ± 0.001	0.0041 ± 0.0001	0.0040 ± 0.0002
Jan-1965		0.192 ± 0.001		0.0039 ± 0.0001
Feb-1965		0.190 ± 0.001		0.0041 ± 0.0001
Mar-1965		0.189 ± 0.001		0.0038 ± 0.0001
Apr-1965		0.186 ± 0.001		0.0039 ± 0.0001
May-1965		0.183 ± 0.001		0.0043 ± 0.0002
Jun-1965		0.181 ± 0.002		0.0037 ± 0.0001
Jul-1965		0.184 ± 0.001		0.0037 ± 0.0001
Aug-1965		0.184 ± 0.001		ND^b^
Sep-1965		0.190 ± 0.002		0.0041 ± 0.0003
Oct-1965		0.187 ± 0.001		0.0037 ± 0.0001
Nov-1965		0.203 ± 0.002		0.0046 ± 0.0002
Dec-1965		0.181 ± 0.001		0.0035 ± 0.0003
Jan-1966		0.183 ± 0.002		0.0039 ± 0.0001
Feb-1966		0.181 ± 0.001		ND^b^
Mar-1966				
Apr-1966		0.186 ± 0.001		0.0040 ± 0.0001
May-1966		0.175 ± 0.002		0.0034 ± 0.0001

^a^Mean  ±  uncertainty (coverage factor k = 1).

^b^Not detectable.

### ^240^Pu/^239^Pu and ^242^Pu/^239^Pu atomic ratios of atmospheric deposition samples

The ^240^Pu/^239^Pu and ^242^Pu/^239^Pu atomic ratios of the atmospheric deposition samples are reported in Table 1. Comparison of the ^240^Pu/^239^Pu and ^242^Pu/^239^Pu atomic ratios measured in samples collected in Tokyo and Akita during the same month show good correlations between the two locations (Fig. S1; Pearson’s correlation coefficient: *r*^2^ = 0.98 for ^240^Pu/^239^Pu and *r*^2^ = 0.97 for ^242^Pu/^239^Pu), i.e. the Pu isotopic compositions of atmospheric deposition samples were almost the same between Tokyo and Akita.

**Figure 2:**
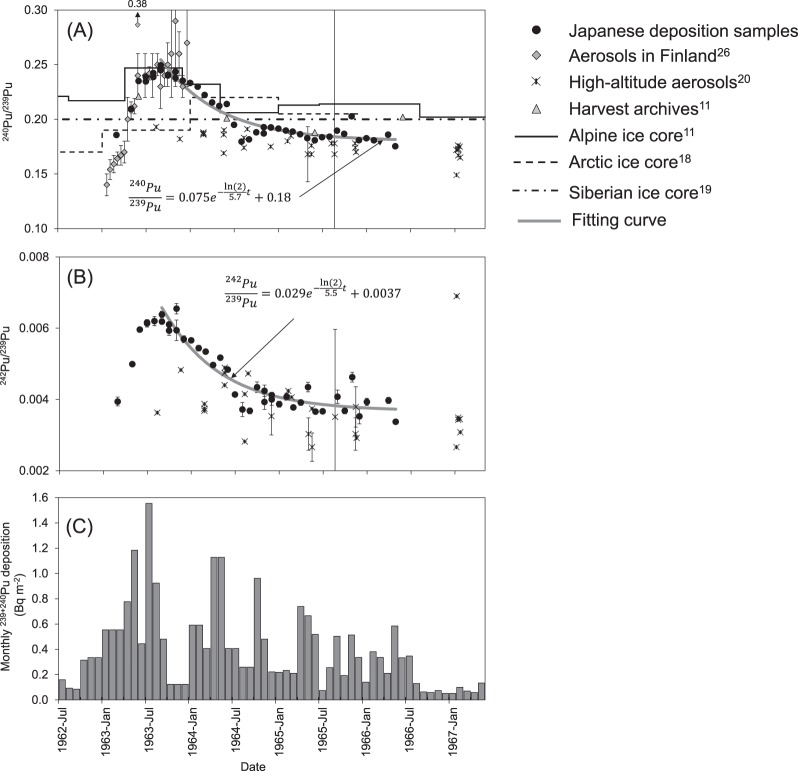
Plutonium atmospheric deposition in Tokyo and Akita, Japan during 1963–1966. (**A**) ^240^Pu/^239^Pu and (**B**) ^242^Pu/^239^Pu atomic ratios of monthly deposition samples collected in Tokyo and Akita. (**C**) Monthly ^239 + 240^Pu deposition flux in Tokyo, from Miyake *et al*.^21^ ^240^Pu/^239^Pu atomic ratios (**A**) are compared to those of aerosol samples collected near ground in Finland^26^, UK harvest archives^11^, Alpine^11^, Arctic^18^, and Siberian Altai ice-core samples^19^. Both ^240^Pu/^239^Pu (**A**) and ^242^Pu/^239^Pu atomic ratios (**B**) are compared to those of high-altitude aerosol samples collected at 35° N^20^. Equations in (**A**) and (**B**) present the fitting curves from temporal variations of the ratios of our deposition samples during September 1963 to May 1966.

Temporal variations of the ^240^Pu/^239^Pu and ^242^Pu/^239^Pu atomic ratios deposited in Tokyo and Akita during March 1963 to May 1966 (Fig. 2A,B) are compared to monthly ^239 + 240^Pu atmospheric deposition fluxes in Tokyo during the same period (Fig. 2C)^21^. From March 1963 (^240^Pu/^239^Pu = 0.186), monthly deposition rapidly increased through June 1963 and then gradually increased to a maximum value of 0.249 in September 1963. The ratio then gradually decreased to 0.180 in August 1964 and remained almost constant with occasional small variations throughout the rest of the study period. The temporal variations of ^242^Pu/^239^Pu were similar to those of ^240^Pu/^239^Pu.

## Discussion

The Pu isotopic ratios in global fallout have been measured in various environmental samples. For comparison with our data, we selected Pu isotopic ratios measured in aerosols^20,26^, plant archives^11^, and ice core samples^11,18,19^ (Fig. 2A), because they represent simple, non-remobilized Pu transport pathways and have similar temporal resolutions. The observed rapid increase of the ^240^Pu/^239^Pu atomic ratio from the end of 1962 to summer 1963 was also observed in aerosol samples collected near the ground in Finland^26^, though measurement uncertainties were relatively large, and variations in late 1963 were substantial. Alpine ice core samples showed similar, but narrower, ^240^Pu/^239^Pu variations as observed in our data, due to their limited temporal resolution. The peak ^240^Pu/^239^Pu atomic ratio observed in 1963 did not appear in Arctic ice core samples^18^ or aerosols collected at high altitudes (4.6–21 km at 35°N)^20^. Rothamsted harvest archive samples collected during 1963–1966^11^ showed values similar to our data.

**Figure 3:**
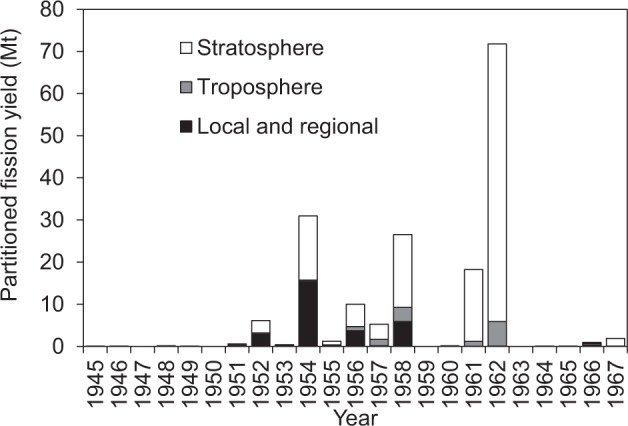
Annual fission yields injected into the stratosphere, troposphere, and local and regional atmospheric regions during 1945–1967^27^.

The fission yields of nuclear weapon testing were compiled and partitioned into tropospheric, stratospheric, and local and regional atmospheric inputs shown in Fig. 3 by the United Nations Scientific Committee on the Effects of Atomic Radiation (UNSCEAR)^27^. Local inputs did not affect Pu deposition in Tokyo and Akita during the study period because of their long distances from proving sites, Hiroshima, and Nagasaki. While there was no atmospheric nuclear weapon testing during 1963, the USA and the former USSR tested their high yield devices during 1961–1962 before the conclusion of the Partial Test Ban Treaty (PTBT) in August 1963. China performed small-scale nuclear testing in 1964 and 1965. In 1966, China and France began testing relatively high yield thermonuclear explosions over 0.1 Mt^27^.

Since the mean residence time of airborne particulates in the troposphere is 30–70 d^23,28^, the tropospheric Pu input from nuclear debris in 1962 was hard to give large effect to the Pu deposition in Japan in March 1963, i.e. 2–3 months after the final USSR tests at the end of 1962. Thus, most of the Pu deposited in Japan in 1963 was derived from the stratosphere. Nuclear debris is introduced into the stratosphere when the detonation yield exceeds 0.1 Mt^29^, though its partitioning between the stratosphere and troposphere depends on the latitude of detonation^25^. Because there were no tests with yields over 0.1 Mt in 1959–1960^27^, and the mean stratospheric residence time of nuclear debris is 1–2 yr^30–33^, the observed increase of ^239 + 240^Pu deposition fluxes and atomic ratios in 1963 must have resulted from high yield nuclear testing conducted during 1961–1962.

Because heavier Pu isotopes are produced in nuclear detonations through successive neutron absorption of ^239^Pu as fission fuel and ^238^U as tamper and fission fuel, the ^240^Pu/^239^Pu atomic ratio produced in nuclear explosions depends on the neutron flux at detonation^14,34^. Because higher yield detonations have higher neutron fluxes, higher ^240^Pu/^239^Pu and ^242^Pu/^239^Pu atomic ratios are expected from higher-yield detonations.

Weapons-grade Pu has very low ^240^Pu/^239^Pu atomic ratios of around 0.07^35^. ^240^Pu/^239^Pu atomic ratios in soil and sediment samples around the Nevada (USA) and Semipalatinsk (former USSR) test sites were reported to be 0.03–0.05^8,36–38^. These low values are the result of the low mean detonation yields of 12 kt (maximum 74 kt) and 63 kt (maximum 1.6 Mt) at the Nevada and Semipalatinsk sites, respectively, despite the large number of atmospheric nuclear tests performed at each site^27^. Such low atomic ratios were also found in soil and sediment samples around Nagasaki, Japan^39–41^ (21-kt yield combat detonation)^27^. In contrast, relatively high ^240^Pu/^239^Pu atomic ratios of 0.28–0.34 were found in soil samples from the Pacific Proving Grounds (Bikini, Enewetak, and Rongelap Atolls in the northern Marshall Islands)^34,39,42^, where 65 atmospheric nuclear tests with a mean detonation yield of 1.7 Mt (maximum 15 Mt) were conducted during 1946–1958^27^. Because the mean detonation yield of 62 tests at the Novaya Zemlya test site (former USSR) during 1961–1962 was 3.5 Mt (maximum 50 Mt)^27^, it is reasonable that Pu from this area had ^240^Pu/^239^Pu atomic ratios similar to or larger than those observed in Marshall Islands soils. Therefore, the high ^240^Pu/^239^Pu atomic ratios observed in Japan during 1963–1964 very likely derived from the high-yield testing at Novaya Zemlya in 1961–1962.

According to UNSCEAR^27^, in 1962, fission-yield partitioned to the upper and lower stratosphere in polar regions (30–90°N including sampling points in this study) were 41.5 and 9.48 Mt, respectively. Thus, significant amounts of Pu with high ^240^Pu/^239^Pu atomic ratios were loaded into the upper stratosphere, whereas older debris with relatively low ^240^Pu/^239^Pu atomic ratios remained in the lower stratosphere.

In polar regions, the half-life of debris removal from the upper to lower stratosphere is 6–9 months, and that from the lower stratosphere to the troposphere is 3–12 months^27^. Thus, the observed increase in ^240^Pu/^239^Pu atomic ratios in early 1963 was due to debris descending through the polar stratosphere and into the troposphere. The gradual decrease of ^240^Pu/^239^Pu atomic ratios after the summer of 1963 resulted from the decreased transport of Pu from the polar stratospheres to the troposphere. We fit the observed ^240^Pu/^239^Pu and ^242^Pu/^239^Pu atomic ratios during September 1963 to May 1966 as follows:

1 $${}^{240,242}\,{\rm{P}}{\rm{u}}/{}^{239}\,{\rm{P}}{\rm{u}}={\rm{A}}\cdot {{\rm{e}}}^{{\textstyle \text{-}}{\rm{l}}{\rm{n}}(2)/{\rm{T}}\cdot t}+{\rm{C}}$$

where T (yr) and *t* (yr) are a half-life of the atomic ratio and a time from September 1963, respectively, and A and C are fitting constants, and we found the half-life (±a standard error) to be 5.6 ± 1.0 months (Fig. 2A) for ^240^Pu/^239^Pu atomic ratio. It is hard to predict the half-life of ^240^Pu/^239^Pu atomic ratio in the atmosphere by using an atmospheric model, because the atomic ratio of each detonation is unknown. Quantitative discussion about the half-life will be the research task in future.

The temporal variations of the deposited ^242^Pu/^239^Pu atomic ratio were similar to those of the ^240^Pu/^239^Pu atomic ratio, i.e. they increased rapidly from March to June 1963, fluctuated throughout the summer of 1963, and gradually decreased until the end of 1964. By fitting the data after September 1963 using Equation (1), we found the half-life of the exponential term to be 5.5 ± 1.1 months (Fig. 2B), very similar to that obtained for ^240^Pu/^239^Pu. It is therefore reasonable that the increased ^240^Pu/^239^Pu and ^242^Pu/^239^Pu atomic ratios from March to June 1963 resulted from high yield nuclear weapon testing during 1961–1962.

Published data on ^242^Pu/^239^Pu atomic ratios are scarce in comparison with ^240^Pu/^239^Pu because of the low concentrations of ^242^Pu. Only high-altitude aerosol data were available at sufficient temporal resolution during the 1960s^21^ to be comparable to our data (Fig. 2B). ^242^Pu/^239^Pu atomic ratios in aerosol samples were lower than those observed in our data during 1963, but they were similar to our data during mid-1964 to 1966. This was also the case for ^240^Pu/^239^Pu atomic ratios, and these inconsistencies may result from differences in sample type, sampling altitude and location, and duration for one sample.

**Figure 4:**
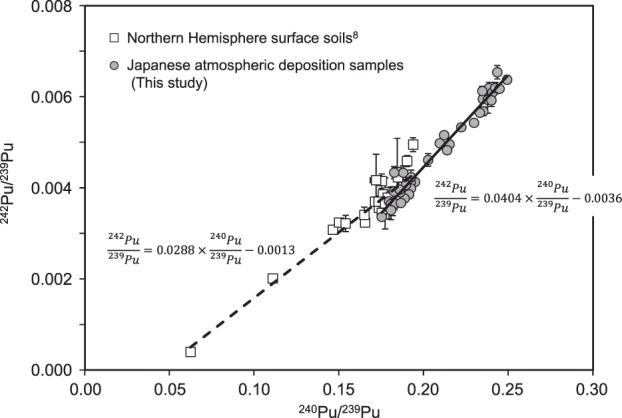
^240^Pu/^239^Pu and ^242^Pu/^239^Pu atomic ratios of Japanese deposition samples during May 1963 to May 1966 compared with those of surface soils collected throughout the Northern Hemisphere^8^. Solid and dash lines are the regression lines of results from our deposition samples and these surface soils^8^, respectively.

We compared the correlation between the ^242^Pu/^239^Pu and ^240^Pu/^239^Pu atomic ratios of our samples to that of surface soil samples collected throughout the Northern Hemisphere in 1970–1971^8^ (Fig. 4). The Pu isotopic ratios of our data were well correlated each other (Pearson’s correlation coefficient: *r*^2^ = 0.97, n = 42).

Although the Pu isotopic ratios of the surface soil samples were also well correlated (*r*^2^ = 0.90, n = 37), the slope of their regression curve was slightly, but significantly (ANCOVA, p < 0.001) gentler than that of our depositional data. Because the surface soil samples were collected in 1970–71^8^, they were affected by Pu deposition on the ground before 1963 and after 1966. Thus, the gentler slope observed for soil samples may reflect the lower mean yield per detonation during the effective sampling period as compared to that during our sampling period.

## Methods

### Pu isotopic analyses

We followed previously established methods^43^ for the decomposition of atmospheric deposition samples and chemical separation of Pu. Briefly, a 1-g aliquot of sample was fused with a mixture of bicarbonate salts and boric acid using a bead sampler (TK-4100, Tokyo Kagaku Co. Ltd., Japan) at 1200 °C for 7 min. The fused sample was then dissolved with 3 M nitric acid, and Pu was separated from the matrix elements using Chelex 100 R resin (Bio-Rad Laboratories, USA). The Pu eluted from the resin was further separated from U using UTEVA resin (Eichrom Industries, USA), because even a trace of U could interfere with ICP-MS Pu measurements. The ^240^Pu/^239^Pu and ^242^Pu/^239^Pu atomic ratios were measured using a MC-ICP-MS (Nu Plasma HR, Nu Instruments Ltd., UK) equipped with twelve ceramic Faraday cups and three full-sized ion counters that can be utilized simultaneously. The ion counters were used for Pu isotopes, and the Faraday cups for other elements. The configuration of ion counters was optimized for the simultaneous detection for ions with *m/z* of 239, 240 and 242; therefore we did not measure ^241^Pu. Plutonium mass bias correction was performed by the external correction technique^44^ using the U isotopic ratios of a multi-element standard solution (XSTC-829, SPEX CertiPrep, USA). We purified a Pu solution from reference material IAEA Soil-6 (International Atomic Energy Agency, Austria) for use as a working standard during Pu isotopic measurements of the atmospheric deposition samples. The Pu isotopic ratios of the atmospheric deposition samples were measured by the sample-standard bracketing method using the working standard Pu solution. The ^240^Pu/^239^Pu and ^242^Pu/^239^Pu atomic ratios we measured for IAEA Soil-6 were 0.191 ± 0.001 and 0.0050 ± 0.0001, respectively. Uncertainties of the atmospheric deposition samples were propagated from the uncertainty of the Pu isotopic ratio of the working standard and the standard deviation of results from repeated measurements. ^238^U can interfere with accurate ^240^Pu/^239^Pu analyses through formation of polyatomic ^238^U^1^H^+^ ions (*m*/*z* 239). The contribution of ^238^U^1^H^+^ ions was corrected using the count rate of ^238^U^+^ ions in the sample and the ^238^U^1^H^+^/^238^U^+^ count ratio of the Pu-free U standard solution. Because the mass concentrations of U in the sample solutions were similar to those of Pu, and the ^238^U^1^H^+^/^238^U^+^ count ratio was ~1.4 × 10^−5^ in our system, the corrections for ^240^Pu/^239^Pu and ^242^Pu/^239^Pu were in the range of 10^−6^ and 10^−8^, respectively, i.e. negligibly small compared to the measured sample ratios.

Unfortunately, there is no standard sample with certified values for Pu isotopic ratio. To validate our analytical method, we analysed two international reference sediment samples (IAEA 368, International Atomic Energy Agency, Austria, and NIST 4354, National Institute of Standards and Technology, USA) and a Japanese reference fallout material produced by the MRI from samples collected at 14 locations throughout Japan during 1963–1979^45^. Our Pu isotopic measurements of these standard samples are presented and compared to results from other researchers in Table 2. The obtained ^240^Pu/^239^Pu atomic ratios agreed well with previous data^9,46–50^, whereas the ^242^Pu/^239^Pu atomic ratios obtained for IAEA 368 varied considerably because of that sample’s low ^242^Pu concentration. It is notable that the relative uncertainties of isotopic ratios for NIST 4354 were larger than those for the present samples in Table 1 because of lower concentration of Pu in measuring solution for the former sample.

**Table 2:** ^240^Pu/^239^Pu and ^242^Pu/^239^Pu atomic ratios measured herein and from literature for IAEA 386, NIST 4354, and Japanese fallout reference material.

Reference material	Atomic ratio		Reference
	^240^Pu/^239^Pu	^242^Pu/^239^Pu	
IAEA 368	0.0337 ± 0.0006^a^	0.00054 ± 0.00009^a^	This study
	0.0347 ± 0.0002^b^	0.0009 ± 0.0006^b^	Jakopič *et al*.^9^
	0.0342 ± 0.0001^b^	0.0003 ± 0.0001^b^	Jakopič *et al*.^9^
	0.04 ± 0.01^b^		Kim *et al*.^47^
	0.0315 ± 0.0004^b^		Donard *et al*.^47^
	0.030 ± 0.004^b^		Donard *et al*.^47^
NIST 4354	0.165 ± 0.003^a^	0.0105 ± 0.0007^a^	This study
	0.153 ± 0.002^b^	0.010 ± 0.001^b^	Jakopič *et al*.^9^
	0.145 ± 0.005^b^		Liao *et al*.^48^
	0.184 ± 0.004^b^		Hrnecek *et al*.^49^
Japanese fallout reference material^c^	0.191 ± 0.003^a^		This study
	0.192 ± 0.004^b^		Zhang *et al*.^50^

^a^Mean ± uncertainty (coverage factor k = 1).

^b^Mean ± 1σ.

^c^Produced by MRI from samples collected at 14 locations throughout Japan during 1693-1679^45^.

## Supplementary information

Supplementary Material
