# Supplementary material for: 240Pu/239Pu and 242Pu/239Pu atom ratios of Japanese monthly atmospheric deposition samples during 1963–1966
Source: Sci Rep. 2019 May 30;9:8105. doi: 10.1038/s41598-019-44352-7 (PMC6543033; doi:10.1038/s41598-019-44352-7)
Supplement: Supplementary file 1 — Supplementary Material [file 41598_2019_44352_MOESM1_ESM.pdf]

$^{240}Pu/^{239}Pu$ and $^{242}Pu/^{239}Pu$ atom ratios of Japanese monthly atmospheric deposition samples during 1963–1966

Yoshihito Ohtsuka[1*], Michio Aoyama[2], Yuichi Takaku[1], Yasuhito Igarashi[3], Michinari Hattori[1#], Katsumi Hirose[4], Shun'ichi Hisamatsu[5]

1 Department of Radioecology, Institute for Environmental Sciences, Rokkasho, Aomori 039-3212, Japan

2 Center for Research in Isotopes and Environmental Dynamics, Faculty of Life and Environmental Sciences, University of Tsukuba, Tennoudai 1-1-1, Tsukuba, 305-8572, Japan

3 Institute for Integrated Radiation and Nuclear Science, Kyoto University, 2, Asashiro-Nishi, Kumatori-cho, Sennan-gun, Osaka 590-0494, Japan

4 Faculty of Science and Technology, Sophia University, Chiyoda-ku, Tokyo 102-8554, Japan. Retired on March 2019.

5 Institute for Environmental Sciences, Rokkasho, Aomori 039-3212, Japan

Department of Psychiatry, Kochi Health Sciences Center, Ike 2125-1, Kochi, Kochi, 781-8555, Japan

* Corresponding author

Tel.: +81-175-71-1452; fax: +81-175-71-1492; E-mail: ohtsuka@ies.or.jp

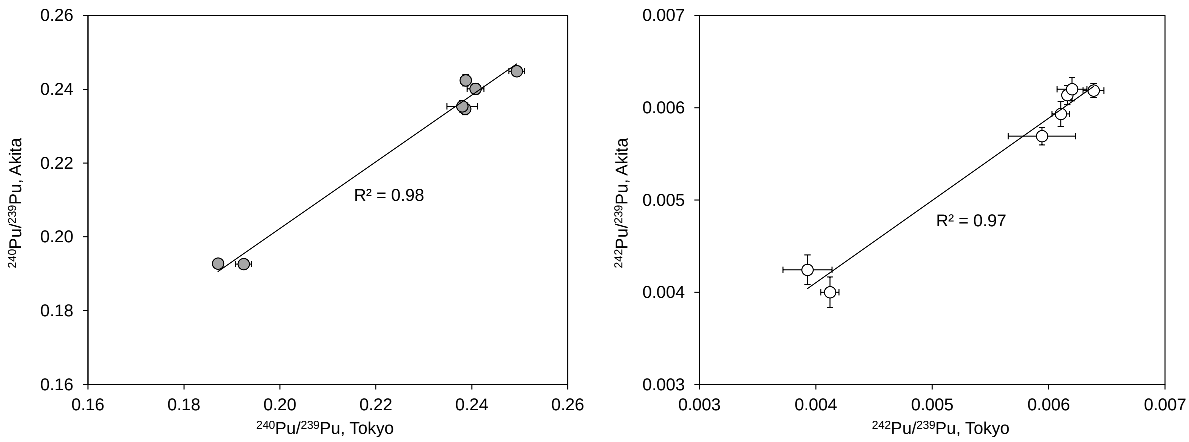

Supplementary Figure S1. Correlation of $^{240}Pu/^{239}Pu$ (left) and $^{242}Pu/^{239}Pu$ atomic ratios (right) between Tokyo and Akita in monthly deposition samples collected in both locations during the same months. Error bars are uncertainties (coverage factor k = 1).
